# Obtaining chicken primordial germ cells used for gene transfer: in vitro and in vivo results

**DOI:** 10.1007/s13353-015-0276-7

**Published:** 2015-03-04

**Authors:** Luiza Chojnacka-Puchta, Dorota Sawicka, Paweł Lakota, Grazyna Plucienniczak, Marek Bednarczyk, Andrzej Plucienniczak

**Affiliations:** 1 Department of Bioengineering, Institute of Biotechnology and Antibiotics, Warszawa, Poland; 2 Department of Animal Biochemistry and Biotechnology, University of Science and Technology, Bydgoszcz, Poland

**Keywords:** Expression vectors, Primordial germ cells, Chicken embryo, Germline chimeras

## Abstract

Recently, several attempts have been made to create a generation of transgenic chickens via chimeric intermediates produced by primordial germ cells (PGCs) transfer. This study aimed to compare the influences of different chicken PGCs isolated from circulating blood (bPGCs) or gonads (gPGCs), purification (ACK, Percoll or trypsin) and transfection methods (electroporation or lipofection) on the expression of transgenes in vitro and the migration of modified donor cells to the recipient gonads. The highest average frequency of pEGFP-N1 plasmid-transfected bPGCs (75.8 %) was achieved with Percoll density gradient centrifugation and electroporation. After ammonium chloride-potassium (ACK) treatment and lipofection, in vitro transgene expression was only detected in 35.2 % of bPGCs. Chimeric chickens were produced from these purified, transfected and cultured cells, and the transgene was detected in the gonads of 44 and 42 % of the recipient embryos that had been injected with bPGCs and gPGCs, respectively. These data confirmed that the combination of PGC purification via Percoll centrifugation and electroporation was an effective method for producing transgenic chickens. Subsequently, we used this method with expression vectors for gene hIFNα 2a/hepatitis B virus surface antigen (HBsAg) under the control of the ovalbumin promoter to generate G0 transgenic chickens. Consequently, we observed that 4.9 % of the hens and 3.5 % of the roosters carried the hIFNα 2a gene, whereas 16.7 % of the hens and 2.4 % of the roosters carried the HBsAg gene, thus undisputedly confirming the exceptional effectiveness of the applied methods.

## Introduction

Since the successes achieved by the first pioneers in the field of transgenic animal production in the early 1980s (Brinster et al. 1981; Gordon and Ruddle 1981), transgenic techniques have developed rapidly to allow the use of various livestock species as alternate methods for producing biologically active substances. To date, recombinant interferon-α 2b, α1-trypsin and monoclonal antibodies have been produced in egg white (Mozdziak and Petitte 2004; McGrew et al. 2004). In avian bioengineering, the chicken primordial germ cells (PGCs) have potential applications. PGCs are specialised germ cells that can transfer genetic information and are, thus, ideal for generating transgenic chickens. To produce transgenic animals, from transferred modified PGCs via chimeric intermediates, we must fully understand the complete mechanisms associated with non-viral gene delivery into embryonic cells, as well as the required chicken cell isolation, purification, culture and transfection conditions. It is also important to address the characteristics of the promoter, enhancer, regulatory and tissue-specific elements in transgenic birds.

In chickens, PGCs have been isolated from the blood (bPGCs) or gonads (gPGCs) of embryos at various stages of development through the use of different purification techniques, including Ficoll, Nycodenz, Percoll density gradient centrifugation, ammonium chloride-potassium (ACK) lysis buffer (Yamamoto et al. 2007), immunomagnetic cell sorting (MACS) and fluorescence-activated cell sorting (FACS) (Mozdziak et al. 2005). These PGCs can be transfected in vitro with avian retroviruses or via non-viral methods and subsequently injected into recipient embryos (reviewed in Song et al. 2010). This study aimed to compare the influences of different chicken PGCs isolation (from circulating blood or gonads), purification (ACK, Percoll or trypsin) and transfection (electroporation or lipofection) methods on in vitro transgene expression. We demonstrate the high percentage of transfected PGCs that express the EGFP by 24-h culturing in a G418-containing medium and their migration to the gonads of recipient embryos. To distinguish the PGCs from other cells, we used periodic acid-Schiff (PAS) staining and immunocytochemistry. The in vitro and in vivo experiments confirmed that PGCs purification with a combination of Percoll centrifugation and electroporation is a potentially powerful tool for putative germline chimera (G0) production. In addition, modification of the strong ovalbumin promoter with regard to own genetic expression constructs might increase the level of protein production in the next generation and, ultimately, in egg whites.

## Materials and methods

### Expression constructs

The pEGFP-N1 plasmid (Clontech Laboratories, Inc., Mountain View, CA, USA) was used as the control DNA for comparing the isolation and transfection methods. To express human IFNα2a/hepatitis B virus surface antigen (HBsAg, GenBank accession no. Z35717), the final vectors were constructed on the pEGFP-N1 backbone (pC_) under the control of the chicken ovalbumin promoter (OVA), including the enhancer and regulatory sequences. The original cytomegalovirus (CMV) promoter sequence was deleted from the pEGFP-N1 plasmid. The chicken ovalbumin promoter region and DNA fragment that encoded interferon α2a were taken from the pOVA and pOVAINT vectors (IBA, Warsaw, Poland; described previously by Bednarczyk et al. 2003) and inserted into pC_ (pC_OVA). The amplified enhancer region 5′ flank of approximately 1,000 bp and the estrogen response element (ERE) sequence were isolated by polyacrylamide gel electrophoresis, digested with *Bam*HI and *Sal*I, and inserted into the pBlueScript SK+ vector. These DNA regions were amplified from 100 ng of total chicken DNA. Specific primers, ERES, EREN, 5OVAB and 5OVAN, are as detailed in Table 1.

**Table 1:** Primers used in this study

Primer	Sequence
ERES	5′aactccgcggctgcagaaaaatgccaggtgga
EREN	5′gaaagcggccgctctagagagagtaagcaacaatct
5OVAB	5′gaaaggatccatgtcagtctgcagaaagagaaa
5OVAN	5′aactgcggccgcattttctcactcactcacctctccaa
GFP1	5′gacgacggcaactacaagac
GFP2	5′gtcacgaactccagcaggac
IFN_F1	5′cagaggaccatgctgactgatc
RevG1	5′gccggtggtgcagatgaactt
HBsAg_F2	5′tcagggcatattgaccacag
RevG2	5′gccggtggtgcagatgaactt

The first and second pairs of primers contained the *Sac*I and *Not*I and the *Bam*HI and *Not*I recognition sequences, respectively. Polymerase chain reaction (PCR) was performed in a total reaction volume of 50 μl that included Buffer 1 and thermostable DNA polymerases from the Expand Long Template PCR System (Roche Diagnostics, Mannheim, Germany). Following an initial 2-min denaturation step at 95 °C, thermostable DNA polymerase was added when the sample achieved a temperature of 90 °C, after which 32 PCR cycles were performed. Each cycle comprised 20 s at 94 °C, 30 s at 58 °C and 2 min at 68 °C. The fragments were ligated into the prepared pC_OVA. preS1/preS2/S HBV was obtained by digesting the pUC-HBV vector (IBA, Warsaw, Poland). The ligation products were transformed into the NEB beta-10 strain of *Escherichia coli* (New England Biolabs, Ipswich, MA, USA). The plasmids used for transfection were isolated with the EndoFree Plasmid Maxi Kit (Qiagen, Venlo, the Netherlands).

### Isolation of PGCs

**Fig. 1:**
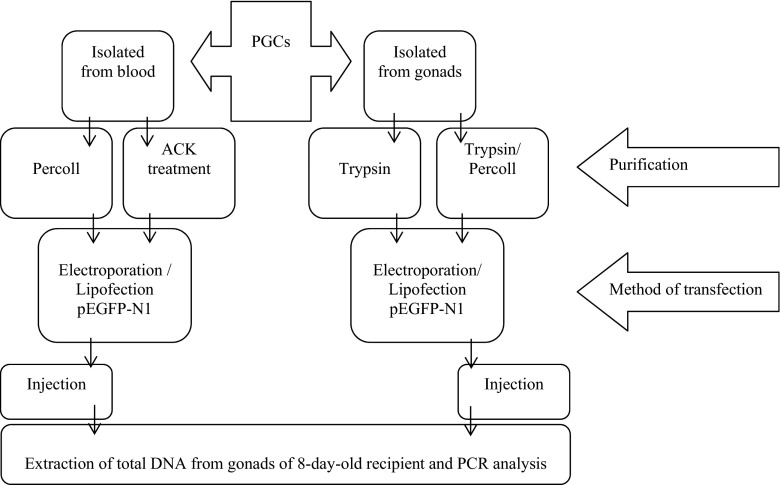
Outline of the in vitro and in vivo studies in which different methods of purification (bPGCs: Percoll, ACK lysis buffer; gPGCs: trypsin digestion, trypsin and Percoll) and transfection (bPGCs and gPGCs: pEGFP-N1 vector electroporation and lipofection) were compared. The most effective combinations for the two types (sources) of cells (bPGCs and gPGCs) were selected for the in vivo experiments

Freshly laid Ross 308 chicken eggs were obtained from a commercial breeding farm (Malec H. Poultry Farm, Góra Kalwaria, Poland) and used as the PGCs donors and recipients. The eggs were incubated at 37.8 °C and the chicken embryos were subsequently staged according to Hamburger and Hamilton (HH staging) (1951). An outline of the in vitro and in vivo studies is presented Fig. 1.

### bPGCs isolation and purification

#### Percoll density gradient centrifugation

PGCs were isolated from embryonic blood at the 14–16 HH stage (50–56 h of incubation) and were suspended in OptiMEM (Gibco Invitrogen Co., Grand Island, NY, USA) supplemented with antibiotics (1 × Penicillin/Streptomycin, Sigma-Aldrich Corporation, St. Louis, MO, USA). One part of the PGCs was purified as described previously (Oishi 2010). Approximately 11.6 × 10^6^ cells were collected, and 3 ml of each Percoll density solution (50 %, 25 % 12.5 % at pH 8.5–9.5; Sigma) were prepared in OptiMEM supplemented with 5 % fetal bovine serum (FBS, Gibco Invitrogen Co., Grand Island, NY, USA). The collected blood containing the PGCs was suspended in OptiMEM with 5 % FBS and layered over the top of the gradient. After centrifugation, the cell layer between the 50 and 25 % Percoll levels was collected and washed twice in OptiMEM with antibiotics.

#### PGCs purification from red blood cells with ACK lysis buffer

A portion of the embryonic blood was treated with 1 ml of ACK lysis buffer (0.15 M NH_4_Cl, 10 mM KHCO_3_, 0.1 mM EDTA). The blood-derived supernatant, which included the PGCs, was centrifuged twice at 2,000 rpm for 7 min at 20 °C. The PGCs pellet was suspended in OptiMEM with antibiotics.

### gPGCs isolation and purification

Gonadal cells were retrieved from the gonads of 6-day-old embryos (28–29 HH). The gonadal tissues were dissociated by pipetting with 0.25 % trypsin (HyClone/Thermo Fisher Scientific, Waltham, MA, USA) at 37 °C and the reaction was stopped by the addition of 10 % FBS. A second group of gonadal cells, after trypsin treatment, was purified via a Percoll density gradient.

### PAS staining, immunocytochemistry and FACS analysis

The cells were fixed in 4 % paraformaldehyde for 5 min and rinsed twice in 1 × PBS (Gibco). The fixed cells were immersed in periodic acid solution for 5 min. After washing with 1 × PBS, the cells were then immersed in Schiff’s solution for 15 min. The PAS-stained bPGCs were observed under an inverted microscope after they were washed twice in 1 × PBS. An anti-SSEA-1 antibody was used to analyse specific surface antigens on the germ cells; briefly, the bPGCs were fixed in 1 % paraformaldehyde-PBS at room temperature for 15 min, after which SSEA-1 expression was determined with an FITC-conjugated antibody (1 μg per 1 × 10^6^ cells) (Santa Cruz Biotechnology, Santa Cruz, CA, USA). FACS was performed using FACS Aria (BD Biosciences, San Jose, CA, USA) to collect a suspension of SSEA-1-positive germ cells.

### Microscopy and cell counting

The viability of the purified PGCs in all experiments was determined with a haemocytometer according to the 0.4 % (*w*/*v*) trypan blue exclusion method (Freshney 1987). The live growing cells, morphological differences and prefixed immunochemically labelled cultures were observed with either bright-field, Nomarski contrast or ultraviolet (UV) light under a fluorescent Eclipse TE2000-E or the Eclipse E800 microscope (Nikon Corporation, Tokyo, Japan). The images were captured with a Nikon or ORCA (Hamamatsu Photonics, Hamamatsu City, Japan) camera coupled to the microscope and processed using the computer-based Nikon EZ-C1 programmable image analyser. A confocal EZ-C1 module and PFS system were used to obtain detailed images of the cells. The emitted fluorescence was collected by filters: FITC (515/30) and PerCp (605/75) after excitation via an argon laser (488 nm).

### PGCs transfection and culture conditions

The cells were purified as follows:

1. bPGCs: ACK treatment or Percoll
2. gPGCs: trypsin or trypsin and Percoll

After purification, the cells were transfected using two methods: electroporation at 200 V and 900 μF for 32 ms or lipofection with Xtreme (Roche) and 20 μg pEGFP-N1. Next, they were seeded into 4-well plates with a density of 5 × 10^5^ cells and over a period of 24 h, the PGCs were grown in OptiMEM I supplemented with antibiotics at 37 °C and 5 % CO_2_ in air. After 24 h, the medium was replaced with OptiMEM I C [C: supplemented with 2 % chicken serum (Gibco), 10 % FBS (Sigma), 20 ng/ml bFGF, basic fibroblast growth factor (Sigma), 9 ng/ml mLIF, murine leukaemia inhibitory factor (Sigma), 5 ng/ml hSCF, human stem cell factor (Sigma), antibiotics and G418 (50 μg/ml, Sigma)]. Every 3 days, the medium was changed.

### Transfer of modified bPGCs and gPGCs to the chick embryos

Transfected bPGCs or gPGCs that had been obtained after purification by Percoll density gradient centrifugation and electroporation of the pEGFP-N1 vector were cultured for 7 days in OptiMEM I C before being transferred into chick embryos at HH stages 14–16. A circular hole was made in the narrow part of the recipient eggshell. Then, 1–1.5 μl of a chicken PGC suspension was injected into the dorsal aorta of the recipient embryo. After injection, the eggshells were sealed and the eggs were returned to the incubator for a total of 8 complete days of incubation at 37.8 °C in 62–65 % relative humidity.

### Observation of the EGFP expression via fluorescent microscope and the analysis of EGFP presence through PCR

The gonads from 8-day-old embryos (control and experimental groups) were dissected and observed after excitation via argon laser (488 nm). Total DNA was extracted from the gonads of the 8-day-old recipient with the DNeasy Blood & Tissue Kit (Qiagen). PCR was performed using the primers GFP1 and GFP2 (Table 1). The 50-μl reaction mixtures contained 10 ng of DNA each, and the reactions were performed as described above. The PCR products were separated on 1 % agarose gel in 1 × Tris-acetate-EDTA (TAE) buffer and visualised by ethidium bromide staining.

#### The preparation of germline chimeric chicken (G0)

The preparation of bPGCs transfected with IFNα2a/antigen HBsAg under ovalbumin promoter was as follows. PGCs were isolated from embryonic blood at the 14–16 HH stage and purified via Percoll density gradient centrifugation. Cells transfected via electroporation with pC-OVAIFN/pC-OVAHBV (IFNα2a/antigen HBsAg) were seeded into 4-well plates with a density of 5 × 10^5^ cells and cultured for 1 week under antibiotic selection in OptiMEM I C.

### Microinjection

After 7 days in culture, 1.5 μl of bPGCs were injected into the recipient embryos (HH stages 14–16) and incubated for 18 days at 37.8 °C and 65 % relative humidity and were rocked at 35° every 2 h except during the last 4 days. The incubation continued until hatching occurred. Untreated fertilised eggs were used as controls.

### DNA extraction and PCR analysis

Samples of semen DNA were obtained from mature G0 roosters and blastodermal DNA cells (BCs) were obtained from hens after fertilisation with Ross 308 male. The DNA was extracted with a cell lysis solution buffer (10 mM Tris–HCl pH 7.6, 10 mM EDTA, 50 mM NaCl and 0.2 % SDS), to which proteinase K had been added at a final concentration of 0.1 mg/ml and was incubated for 2 h at 56 °C. Next, 4 mg/ml of RNAse were added. The proteins were removed by phenol:chloroform (1:1  *v*/*v*) extraction and the DNA was precipitated with ethanol and resuspended in Tris-EDTA (TE) buffer (10 mM Tris–HCl, pH 8.0, 1 mM EDTA).

PCR analyses for the presence of exogenous genes were performed with SuperHotTaq DNA Polymerase (Bioron GmbH, Ludwigshafen, Germany) on 30-ng DNA samples. The primers were IFN_F, RevG, HBsAg_F and RevG (Table 1). The PCR amplifications were performed for 30 cycles. The reaction products were loaded onto an 8 % polyacrylamide gel for molecular sizing. Genomic DNA from an untreated rooster was used as the negative control.

### Statistical data analysis

The statistical analysis was performed with the assistance of the SAS 9.2 software package (2010). To compare the effectiveness of the two purification methods and two cell transfection methods, a two-way analysis of variance for an unbalanced set-up was used (procedure: GLM, III type of quadrant sums). A detailed analysis of the simple effects was performed with Tukey’s honest significant difference (HSD) procedure for an individual classification based on a combination of the purification and transfection methods at a significance level of α = 0.05 and confidence level of 1 − α = 0.95. The confidence ranges for the mean results were established at the same level of confidence (procedures: GLM and MEANS). In the quantitative analysis of the interactions between the purification and transfection methods, the significance tests and confidence ranges (1 − α = 0.95) were applied for the appropriate contrasts. The preliminary results analysis demonstrated that the frequently recommended transformation of fractions (Sokal and Rohlf 1995) was practically meaningless in the case of the analysed data; therefore, it was not used in the presented detailed comparisons.

## Results

### Characterisation of PGCs

**Fig. 2:**
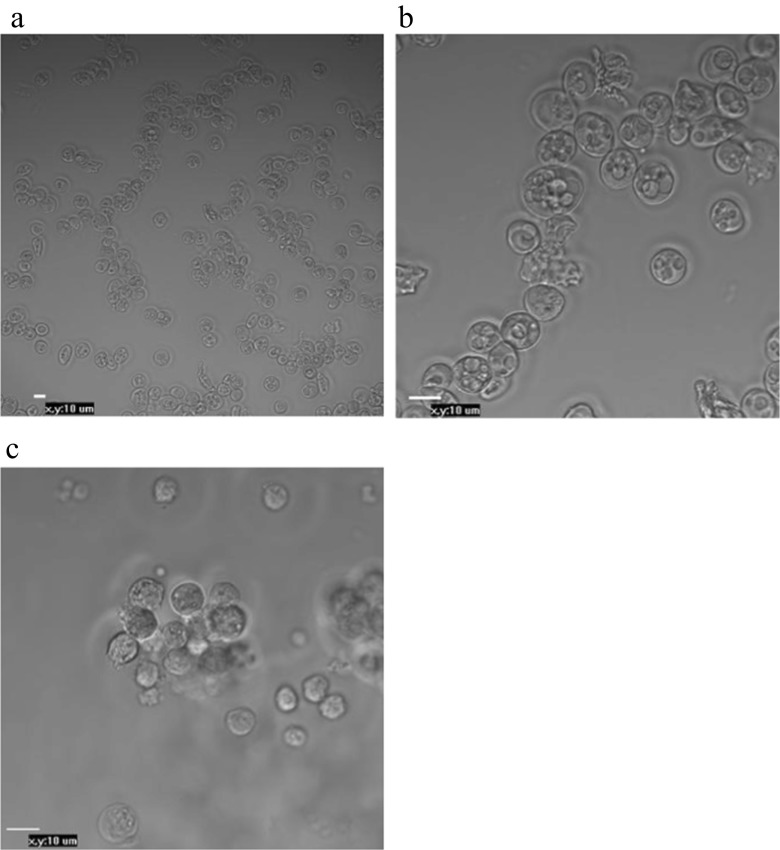
Morphological observations of PGCs that were isolated from the blood of embryos (stages 14–16 HH) and gonads of embryos (28–29 HH), purified and cultured for 24 h. bPGCs collected from between the 25 and 50 % Percoll dilution layers (**a**) (20× magnification). bPGCs after ACK buffer treatment (**b**). gPGCs are shown after trypsin digestion, Percoll purified and cultured for 24 h (**c**) (40× magnification)

**Fig. 3:**
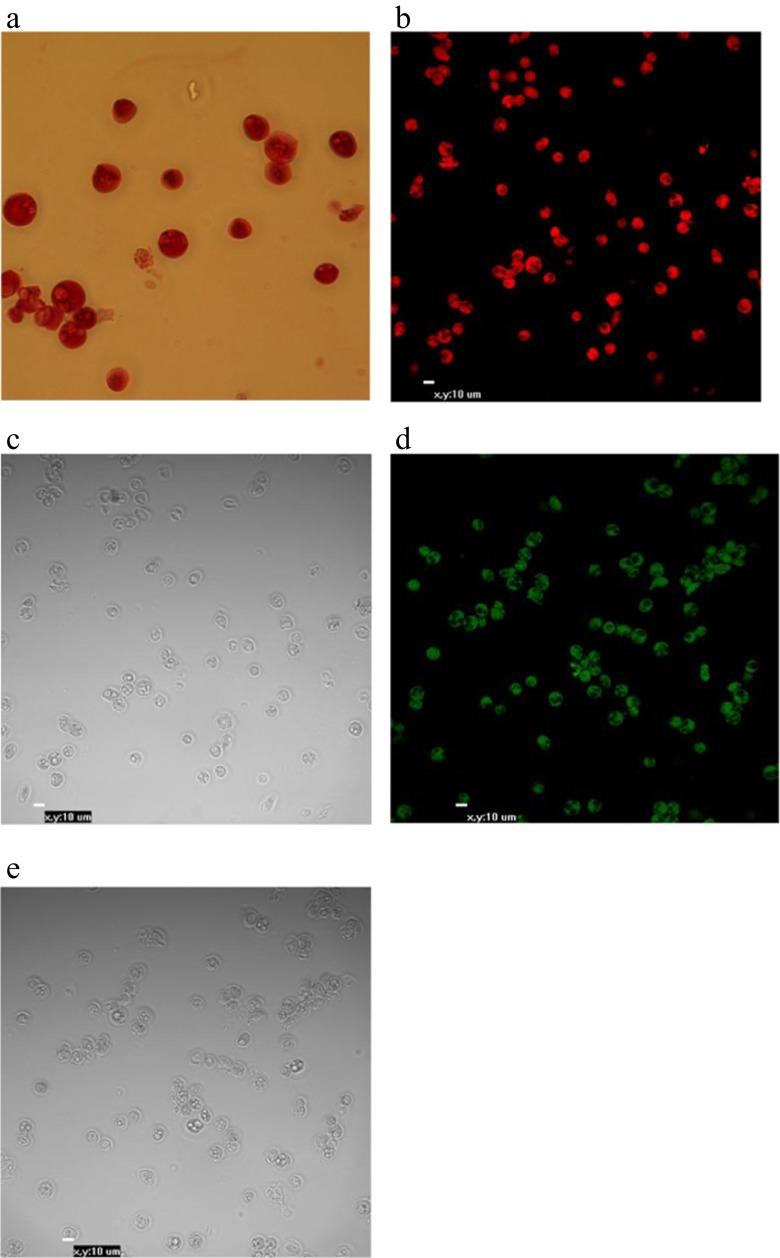
PAS histochemistry indicating the presence of glycogen vesicles and red-stained chicken PGCs (**a**) (40× magnification). Immunofluorescence staining results for the specific cell surface antigen SSEA-1; chicken blood PGCs were found (stages 14–16 HH) to express SSEA-1. Anti-SSEA-PerCp-positive cells are shown in (**b**) and brightness (**c**) and anti-SSEA-1-FITC-positive cells are shown in (**d**) and brightness (**e**) (20× magnification)

**Fig. 4:**
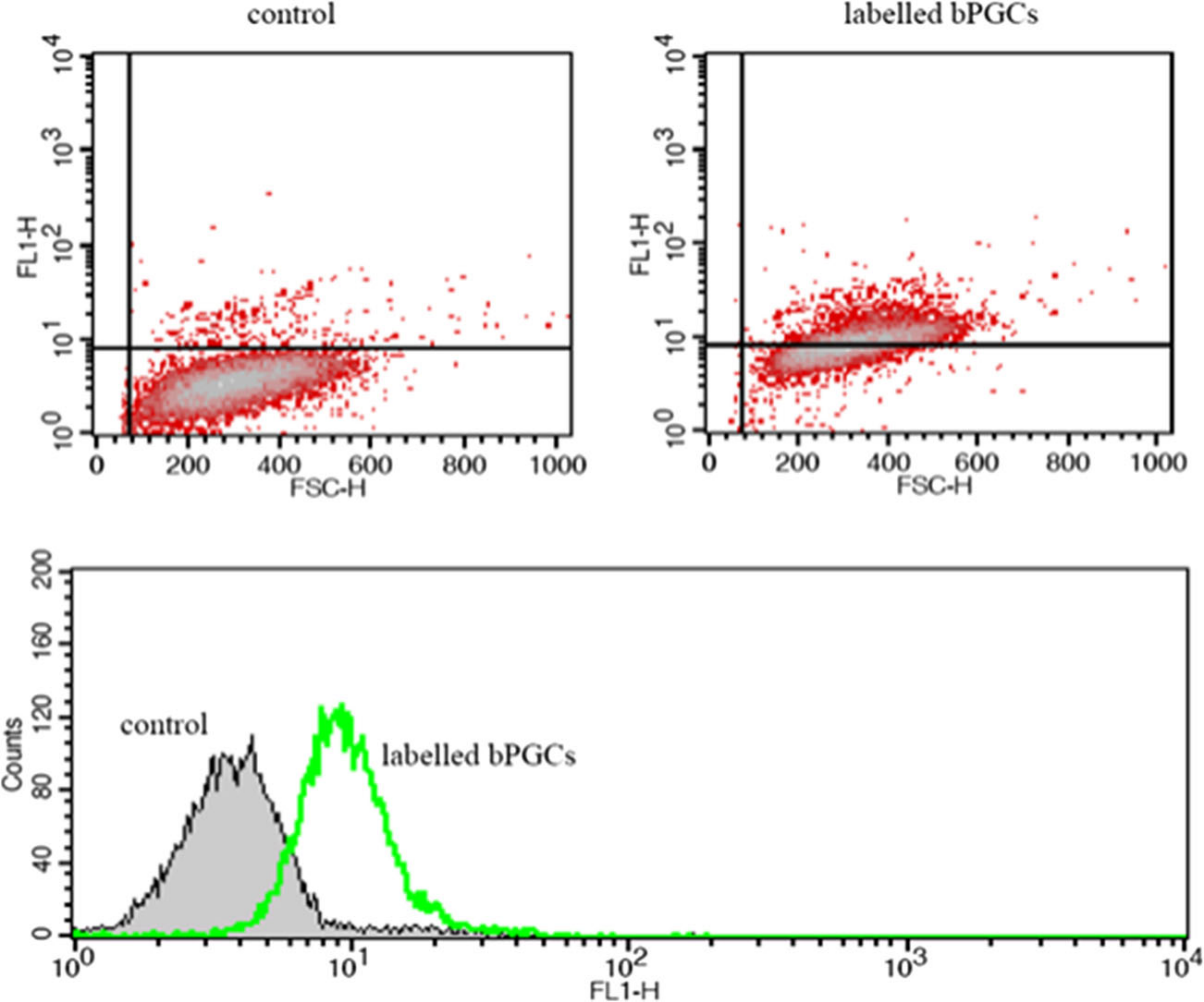
FACS analysis of Percoll-purified bPGCs labelled for SSEA-1-FITC expression. Flow cytometry analysis showed an expression rate of 64 %

This study was based on technology that incorporated non-viral methods applied to modified PGCs to generate germline chimeras. The migration and movement of PGCs through the bloodstream in chicken embryos enabled us to collect these cells from embryonic blood and inject modified PGCs into recipients. Additionally, gonadal PGCs from 6-day-old embryos could proliferate in culture and migrate to the germinal ridge in the recipient. The assessment of PGCs isolation and purification is illustrated in Fig. 2. PGCs purification enabled the production of a homogeneous suspension of viable PGCs (Fig. 2a, c), compared with those obtained with ACK-treated PGCs (Fig. 2b) suspensions. We could also confirm the identities of the PGCs with PAS staining (Fig. 3a) and immunocytochemical methods (Fig. 3b, d). PGCs purified via Percoll centrifugation and labelled with anti-SSEA-1-FITC were found to have an SSEA-1 expression rate of 64 % as determined by FACS (Fig. 4).

### Comparison of the different purification and transfection methods

**Fig. 5:**
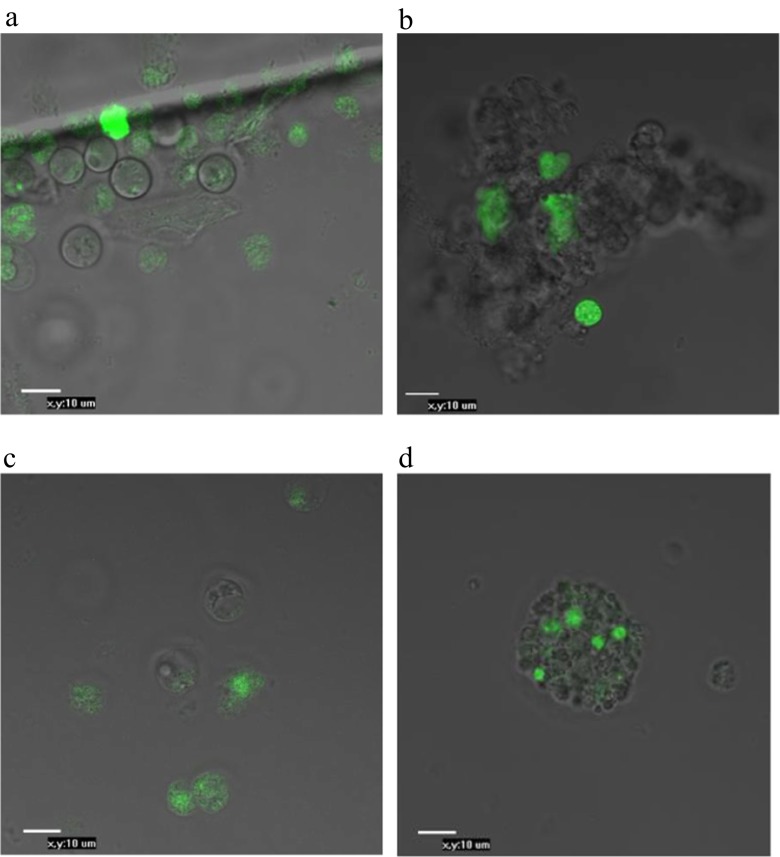
Results of a culture of bPGCs/gPGCs subjected to G418 selection 7 days later (**a**, **b**) and few positive EGFP bPGCs/gPGCs remain after 14 days (**c**, **d**) post-transfection with the pEGFP-N1 vector. EGFP protein-expressing cells were observed on the different surfaces of the specimens (40× magnification)

To study the effects of the purification and transfection methods, the pEGFP-N1 plasmid was used. Cells expressing EGFP are obvious 7 days after transfection (Fig. 5a, b). After 14 days in culture and G418 selection, few fluorescing cells were evident (Fig. 5c, d).

### Assessment of bPGCs

**Fig. 6:**
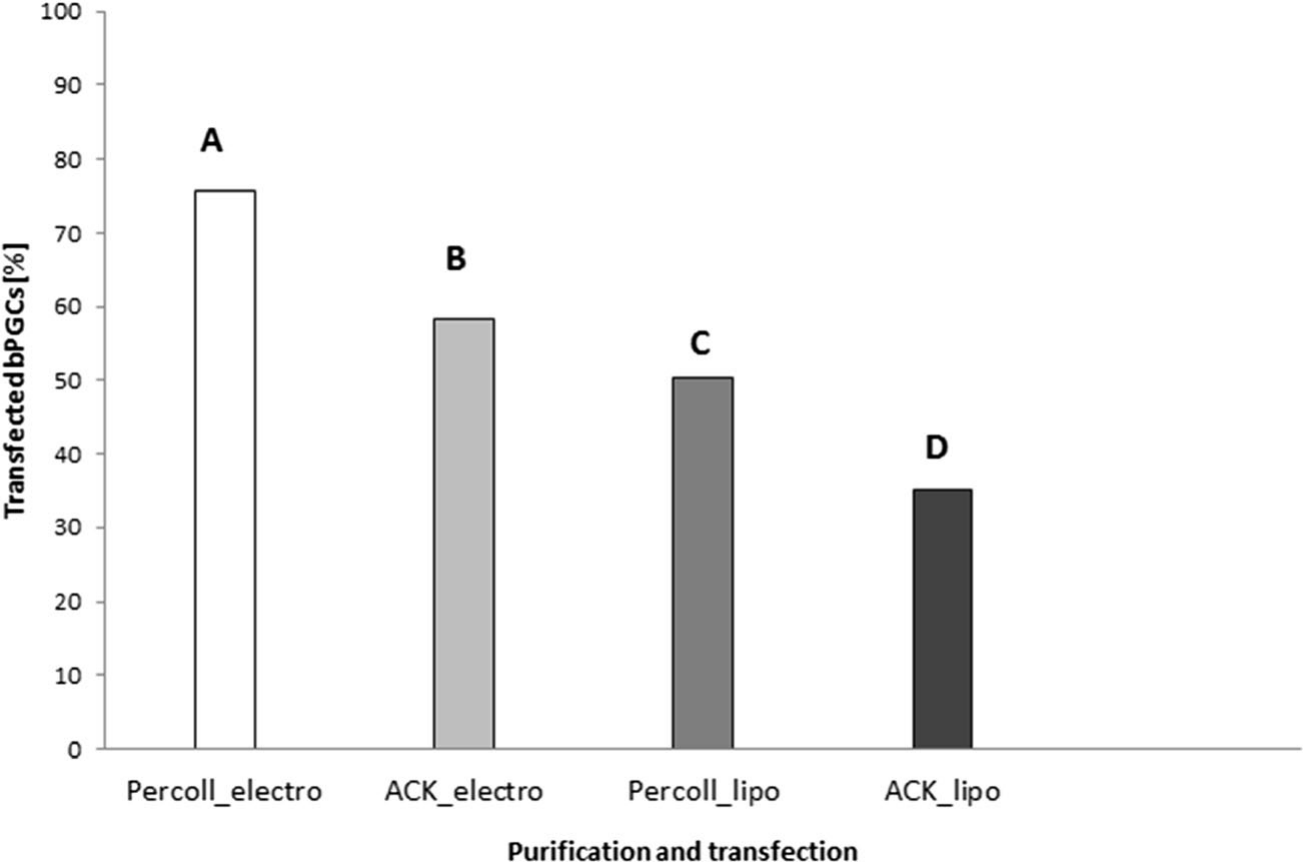
The impacts of the purification and transfection methods on the percentage of transfected bPGCs. The different letters (*A*–*D*) indicate statistically significant differences at a level of α = 0.05

The effectiveness of the use of two purification methods (Percoll or ACK) and two transfection methods (electroporation or lipofection) on the bPGCs was assessed by trypan blue staining after the cells had been cultured to G418 selection 24 h later. The highest mean percentage of transfected bPGCs was 75.8 %, and this level of transfection was acquired by purifying the cells via Percoll centrifugation followed by electroporation [95 % confidence interval (CI): 73.5–78.1 %; Fig. 6]. The combination of lysis in ACK buffer with lipofection yielded the lowest percentage of transfected cells (35.2 %; 95 % CI: 31.4–39.1 %).

### Assessment of gPGCs

**Fig. 7:**
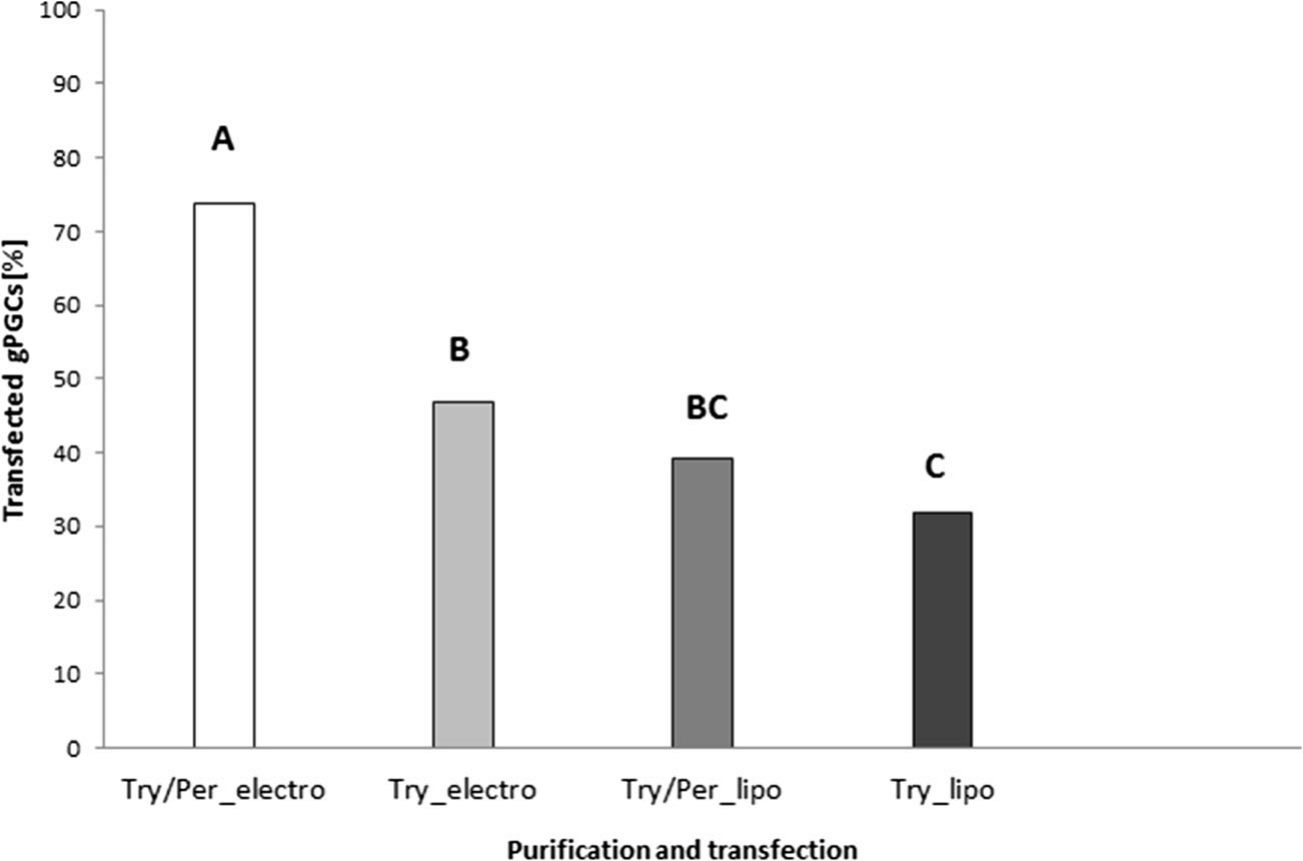
The impacts of the purification and transfection methods on the percentage of transfected gPGCs. The cases marked with the same letter did not statistically significantly differ at a level of α = 0.05

The effectiveness of the use of two purification methods (trypsin digestion with or without Percoll gradient centrifugation) and two transfection methods (electroporation or lipofection) on gPGCs was assessed by trypan blue staining after the cells had been cultured to G418 selection for 24 h later. The results are shown in Fig. 7. Of the gPGCs, the highest mean percentage of transfected cells was 73.8 % (95 % CI: 70.2–77.4 %); this was acquired by cell purification with both trypsin digestion and Percoll gradient centrifugation followed by transfection via electroporation. The most unfavourable set-up that resulted in the lowest mean percentage of cells (31.8 %; 95 % CI: 30.6–32.9 %) was purification via trypsin digestion followed by transfection via lipofection.

### Results of the transfer of modified bPGCs/gPGCs to chicken embryos

**Fig. 8:**
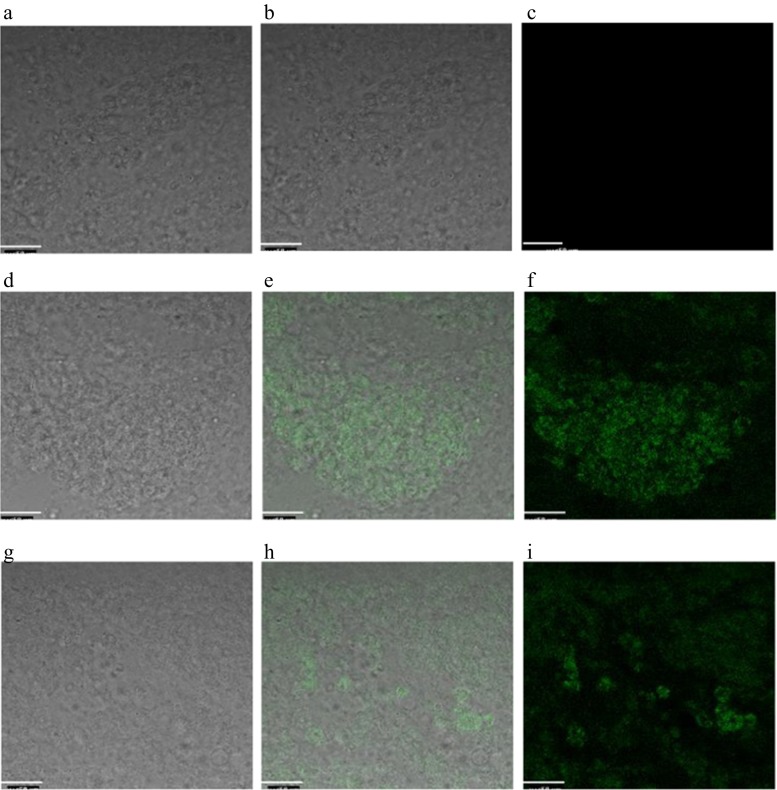
EGFP^+^ gonad cells dissected from 8-day-old embryos (specimen of mashed gonads) (**d**, **e**, **f** and **g**, **h**, **i**). The mashed gonads from 8-day-old untreated chicken embryo (negative control) (**a**, **b**, **c**)

**Fig. 9:**
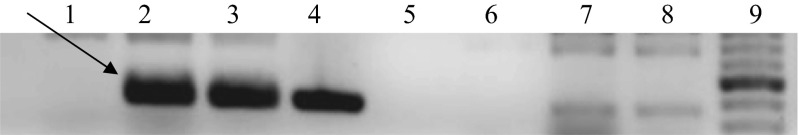
Genomic PCR analysis of recipient embryos after bPGCs transfection (1 % agarose gel). *1*–*3*, *7*, *8* genomic DNA isolated from gonads; *4* control DNA: pEGFP-N1; *5* control mix PCR; *6* total DNA from hen blood was used for the negative control; *9* ladder indicates size of fragments, narrow indicates fragment EGFP (~407 bp)

**Fig. 10:**
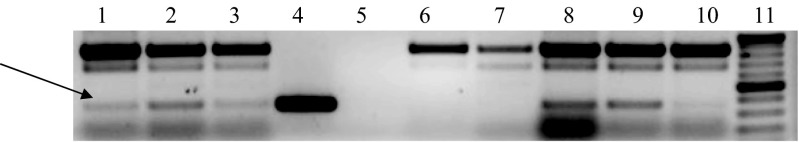
Genomic PCR analysis of recipient embryos after gPGCs transfection (1 % agarose gel). *1*–*3*, *7*–*10* total DNA isolated from gonads; *4* control DNA: pEGFP-N1; *5* control mix PCR; *6* total DNA from hen blood was used for the negative control; *11* ladder indicates size of fragments, narrow indicates fragment EGFP (~407 bp)

The injected embryos were characterised by their high viability rates (87–88 %). Table 2 shows the number of groups and the percentages of live and dead recipient (bPGCs/gPGCs) embryos. Figure 8 illustrates gonad cells dissected from 8-day-old (34 HH) embryos emitting GFP fluorescence after excitation via an argon laser (488 nm). The PCR analysis detected the EGFP gene in the gonads of 44 % of the embryos after injection of the modified bPGCs and 42 % of the recipients after injection of the modified gPGCs (Figs. 9 and 10).

**Table 2:** Viability of recipient embryos after the injection of modified PGCs

Experimental group	Total number of embryos	Percentage of live embryos	Percentage of dead embryos
Injected embryos with bPGCs	26	88.5	11.5
Injected embryos with gPGCs	24	87.5	12.5
Embryos with window for injection	10	80.0	20.0
Control	15	100	0.0

### Analysis of germline chimeric chickens (G0)

Chimeric chickens were produced after injecting the modified PGCs with pC-OVAIFN/pC-OVAHBV. The hatch rate of the injected recipient embryos was 68.3 %. DNA isolated from the BCs and sperm were assessed by PCR to reveal that 4.9 and 16.7 % of the transgenic hens and 3.5 and 2.4 % of the transgenic roosters carried the hIFN α2a gene and HBsAg antigen, respectively.

## Discussion

The generation of transgenic chickens with PGCs, including the isolation and injection of these cells into the recipient embryos, production of germline chimeras and use of the chimeras to create transgenic birds through breeding programmes, is a complex process requiring an appropriate strategy. Some reports have compared the individual procedures that were included in our own research. For example, we can reference comparisons between the cells’ origins (Kim et al. 2005), various methods of cells isolation (Mozdziak et al. 2005; Motono et al. 2010) and procedures for in vitro culture (Macdonald et al. 2010) or methods of transfection.

Our research focused on three main areas. First, it addressed PGCs isolation by indicating the effective purification methods and defining the effective transfection methods with a marker gene construct. Second, it assessed EGFP gene expression both in vitro and in vivo. Third, it addressed the construction of tissue-specific vectors under the control of the ovalbumin promoter and its surrounding enhancer sequences to transfection of the PGCs that were subsequently used to generate putative germline chimeras (G0).

During the early stages of chicken development, PGCs move to the blood and migrate to the germinal ridges (future gonads), where they accumulate as gonadal germ cells (Motono et al. 2010; D’Costa et al. 2001). Previous publications have provided information regarding the numbers of PGCs at subsequent developmental stages. Approximately 30 differentiating cells can be identified while PGCs are migrating through the embryonic cardiovascular system in the X stage of early development (Eyal-Giladi and Kochav 1976). During the subsequent germinal crescent stage, 200–250 cells can be observed (Tsunekawa et al. 2000) and during stage 31 HH (31 HH is at the beginning of the seventh day of incubation), more than 1000 cells are present (Zaccanti et al. 1990). These data illustrate the difficulties associated with determining the number of PGCs that would facilitate further manipulation. The application of modern isolation techniques (Mozdziak et al. 2005; Motono et al. 2010), such as MACS and/or FACS, has yielded some progress in this area. Another method that led to an increase in the number of transfected cells was Percoll gradient centrifugation to fully remove the morphotic elements of blood, debris and dead and defective cells (Matás et al. 2011). Consequently, a uniform viable PGCs population was acquired for further manipulation. In our research, using PAS staining, FACS and an anti-SSEA-1 antibody, the presence of bPGCs was confirmed after purification by Percoll centrifugation. Oishi (2010) indicated that the purification of chicken PGCs on a Percoll gradient increased the effectiveness of both transient and stable transfection.

To demonstrate the migratory activities of the PGCs, we provide efficient and useful in vitro and in vivo techniques for PGCs, although the expression was transient. These cells were purified via Percoll centrifugation and then transfected with EGFP and injected into recipient embryos. The two methods of transfection, electroporation and lipofection, were compared to optimise PGCs transfection. After Percoll gradient centrifugation, the cells were observed to be very healthy (Fig. 2a, c), with no dead or damaged cells at any of the stages. The pEGFP-N1 vector was used for both the electroporation and lipofection experiments. The cells were cultured in a selective medium containing G418; this allowed us to nearly precisely define the percentages of the positively transfected cells resulting from the applied transfection methods. The cells that were not successfully transfected with the neomycin resistance cassette (Neo^r^)-containing pEGFP-N1 plasmid did not produce the enzyme to digest G418 and consequently died. There are several factors which have a huge impact on the sensitivity of the cell population to antibiotics. These include not only the rate of the cell division but also the baseline level of toxicity. We applied the lowest but functional rate of G418 in the cultured media after transfection. The highest mean percentage of transfected bPGCs was 75.8 % (95 % CI: 73.5–78.1 %; Fig. 6), which was achieved using a combination of purification via Percoll centrifugation and transfection via electroporation. The positive results obtained with Percoll purification and electroporation were evident when compared with the results of ACK buffer purification and lipofection (≤9 %). After using a commercial lipofectant, we observed slow cell division in the cultures, which indicated the possible presence of toxic substances. Additionally, the complete removal of the buffer during ACK lysis was difficult and, consequently, we observed that both the morphotic blood elements and the living PGCs were digested.

Furthermore, bPGCs that had purified via Percoll, transfected by electroporation and cultured in selective medium were injected into the recipient embryos; these embryos were left to develop further. We proved that PGCs following transfer to the recipient embryos did not lose their abilities to colonise the gonads. The PCR analysis of isolated embryonic gonadal DNA assesses the presence of the vector pEGFP-N1 sequence at the rate of 44 %. Using Percoll gradient centrifugation, we increased the number of living cells and, thereby, increased the uptake of foreign DNA.

As mentioned in previous publications, the injected and modified gPGCs can migrate to the recipients’ gonads (Kagami et al. 1995; Hong et al. 1998), similarly to the bPGCs collected from blood. Therefore, we designed the in vivo gPGCs experiment with the intent to inject these cells into embryos, thus requiring the gPGCs to be purified by Percoll centrifugation and transfected with EGFP. In our research, the highest mean percentage of transfected cells was 73.8 % (95 % CI: 70.2–77.4 %) after trypsin digestion, Percoll gradient purification and electroporation (Fig. 7). For the gPGCs, the results of this combined method were the most positive and yielded at least 9.5 % more transfected cells when compared with cells that were treated with trypsin alone. The following factors had a significant impact on the transfection method efficiency and played key roles in ensuring the success of this process: properly adjusted electroporation parameters, the applied medium, the cell type and the selected equipment (van de Lavoir et al. 2006). In principle, for non-adherent cells, electroporation is the proper nucleic acid transfection method rather than the commercially available synthetic lipid-based transfection reagents. We confirmed the abilities of transfected PGCs to migrate to and colonise gonads based on the observation of EGFP^+^ PGCs in mashed gonads (Fig. 8) and PCR. The PCR analysis of the recipient embryonic gonads, which allowed the identification of EGFP gene transfection, demonstrated the comparable effectiveness of the transfections of both bPGCs and gPGCs, in response to which modifications were observed in 44 and 42 % of the gonads, respectively (Figs. 9 and 10). Thus, the presence of exogenous DNA in the gonads of embryos does not necessarily indicate the stable germline in chickens (Naito et al. 2007).

Few reports have described the successful use of non-viral methods and solutions are required for various associated problems. To maximise germline transmission, we modified our cell-isolation method to increase the purity of PGCs. Percoll density gradient centrifugation of purified chicken cells was used by Oishi (2010) and this method was also found to increase the efficiencies of both transient and stable DNA transfection of chicken PGCs. We understand how combinations of proliferation factors can limit or increase the PGCs growth in culture. Macdonald et al. (2010) and Choi et al. (2010) have shown conclusively that bFGF is essential for the survival of chicken PGCs in culture. We cultured transfected PGCs in medium C: supplemented bFGF, mLIF and hSCF. After 14 days of PGCs culture, few fluorescing cells were observed (Fig. 5). This suggested that stable integration of the EGFP into the PGCs chromosome is required for its expression.

We recently demonstrated (Chojnacka-Puchta et al. 2012) that the introduction of an ovalbumin-modified, GFP-expressing base vector into cultured chicken oviduct epithelial cells yielded strong fluorescence. This finding allowed us to create identical, final, tissue-specific constructs of pC_OVAHBV and pC_OVAIFN. These constructed vectors could potentially be used to generate transgenic chickens. The simplest solutions could exert large impacts on commercial-scale protein production. The accuracy of our promoter–enhancer combination allowed us to design expression vectors that, when used to modify PGCs, could also be used to generate transgenic chickens. In accordance with this idea, we created constructs that included the genes encoding IFNα2a and the hepatitis-associated cell surface proteins preS1 and preS2/S under the control of the ovalbumin promoter and strengthening sequences, which included the chicken oviduct-specific and enhancer-like (COSE) region and ERE. An analysis of the current publications allows us to state that, to use the potential generated by PGCs, a construct with the maximum expression potential should be designed. The present results support the idea that transcriptionally quiescent germ cells, which are prone to switching off transgene expression (Seydoux and Braun 2006), require suppressors or enhancers to maximise cytoplasmic export and stabilisation of the mRNA transcript (Seo et al. 2010). The latest research has proven that the perfect tool for reducing epigenetic silencing is the ‘cut and paste’ transposon (Macdonald et al. 2012; Park and Han 2012). However, the use of the transposon miniTol2 in the directed transfection of in vivo PGCs has been suggested by Tyack et al. (2013) to fully eliminate the difficulties related to the PGCs culture and retrovirus use. It will be necessary to analyse this method further and to determine, for example, the impact of strengthening sequences such as the central polypurine track element on the expression of the analysed protein in epithelial cells from the chicken oviduct (Jung et al. 2011).

**Fig. 11:**
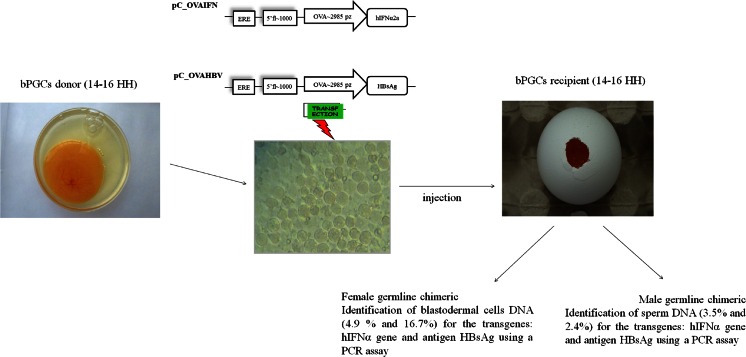
Outline of the final in vitro and in vivo studies; effectiveness of the applied methods

This PGCs technology (Fig. 11) has been successfully applied in the project titled “The germ cells modification and obtaining of birds carrying the genes of therapeutic proteins” (National Center for Research and Development, grant no. N R12 0110 10). The basic strategy of this grant is to obtain the generation of transgenic birds that synthesise recombinant therapeutic protein to a secretory tissue in the oviduct of laying hens. In this project, we used a non-viral ovalbumin promoter system to modify the PGCs to express the human IFNα gene and HBsAg. The generated G0 birds were screened in a PCR assay and the following expression percentages were obtained for the transgenes hIFNα2a and HBsAg, respectively: hens DNA (4.9 and 16.7 %) and roosters DNA (3.5 and 2.4 %). Test mating G0 (data not shown) carrying the hIFNα2a gene allowed to obtained germline transmission G1 at levels of 6.7, 5 and 2.3 %, depending on three individuals of roosters. In case of the HBsAg antigen, the germline transmission rate after mating one rooster with seven hens was at the level of 34.2 %. In doing so, we proved that donor-derived functional gametes could be produced from the putative chimeric chickens. In future studies, we will collect eggs laid by the transgenic G1 and G2 hens and test for the presence of recombinant proteins in the egg whites using an enzyme-linked immunosorbent assay (ELISA).
